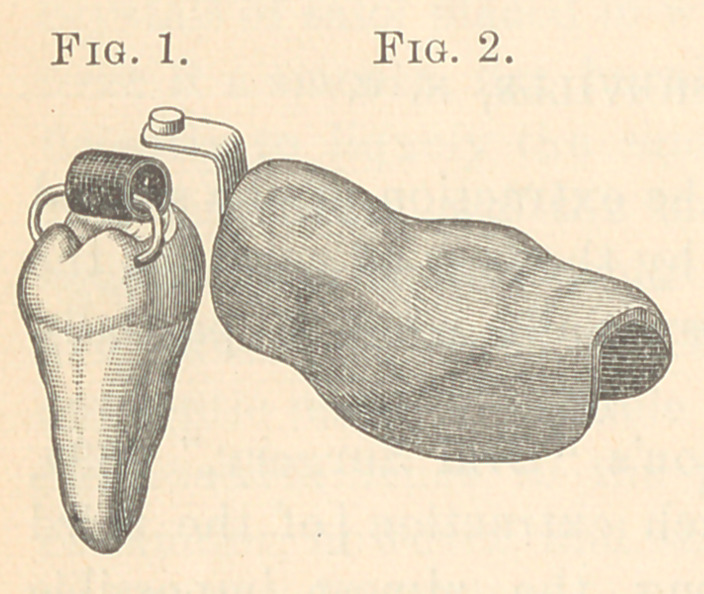# Impacted Third Molars

**Published:** 1895-08

**Authors:** J. W. Foreman

**Affiliations:** Asheville, N. C.


### IMPACTED THIRD MOLARS.

BY DR. J. W. FOREMAN, ASHEVILLE, N. C.

The text-books are still teaching the extraction of the second molars for the relief of trouble caused by the lack of room for the proper eruption of the third molar, especially when it partially erupts in a horizontal position.

The last (sixth) edition of Garretson's "Oral Surgery," 1894, says, in discussing this condition, "Such extraction [of the third molar], however, is occasionally among the almost impossible things. A tooth so affected will not infrequently have but a point not larger than the head of a pin erupted. In these cases the best thing to be done is to *take out the adjoining molar.*" (See p. 859.)

I have never seen a case where this was either necessary or justifiable. It is not necessary because, if the third molar is through the gum at all, with proper appliances it can be brought far enough up to be grasped with the forceps and removed. It is not justifiable because in the vast majority of instances we deprive the patient of a very valuable tooth, the extraction of which is in no degree necessary; and, as a rule, the third molar proves of but little value, tipping forward if not erupted in a horizontal position, thus producing a practical loss of two teeth instead of one.

From my observation and experience I can conceive of no case, where the tooth is through the gum at all, or even above the edge of its socket, in which it would be anything but *bad* practice to extract the second molar, except where inflammation is so great that it would be dangerous to delay extraction long enough to bring the offending tooth up within the grasp of the forceps, or the possible case where there might not be strength enough in the tooth to resist the force necessary to dislodge it. Even where the inflammation is severe it will subside quickly, as a rule, after the appliance for elevating the tooth is in place, as this at once prevents the bruising and irritating of the overlying soft parts, which causes the trouble. When the tooth is far enough erupted to allow a firm hold with the forceps there is seldom need for anything more than to grind or file away the tooth sufficiently to free it from contact with the second molar, or to separate them as for filling, that the tooth may be drawn away from the ramus. Should the tooth present horizontally, or be so imperfectly erupted as to be beyond the reach of the forceps, an apparatus to elevate it is needed.

The appliance used in dealing with the last case is shown in Fig. 2. It is a vulcanite cap to cover the two molars and second bicuspid, with a gold arm bent so as to project over the tooth to be lifted, and as far above it as the upper jaw will permit. The remainder of the apparatus consisted of a gold-wire staple with the ends bent the second time so as to form an enclosed, long link, the ends of which were pinched together into holes drilled in the buccal and lingual sides of the tooth, and a piece of rubber tubing to connect this staple with the projecting arm of the cap.

FIG. 1.

FIG. 2.

The idea was first to get the force from a screw, but it was found that the patient could not manage that, and the elastic was substituted. The cap was removed, cleaned, and replaced once a day by the patient.

This tooth was placed horizontally, the grinding surface impinging upon the distal root of the second molar; the distal surface, the only part uncovered, less than one-fourth of the circumference of the tooth. The gum had to be cut away on both sides to get low enough down to drill the holes, the discolored part between the holes being all that was exposed. Fig. 1 shows tooth, staple, and elastic.

Ten days sufficed to lift the tooth enough to permit fairly easy extraction. A severe otalgia, from which the patient had suffered for weeks, disappeared almost as soon as the tooth began to move, and, with the exception of a slight attack the day after extraction, has never returned.

The difficulties in extracting these teeth are often very great, it is true, but it has never been my misfortune to find them so great as to force me to sacrifice the second molar.

## Figures and Tables

**Fig. 1. Fig. 2. f1:**